# TMT-based quantitative proteomics analysis reveals the role of Notch signaling in FAdV-4-infected LMH cell

**DOI:** 10.3389/fmicb.2022.988259

**Published:** 2022-09-15

**Authors:** Yujuan Niu, Zhiyang Liu, Mengyu Wang, Ke Du, Kaihui Chang, Yonghe Ding

**Affiliations:** The Biomedical Sciences Institute of Qingdao University (Qingdao Branch of SJTU Bio-X Institutes), Qingdao University, Qingdao, Shandong, China

**Keywords:** proteomics, FAdV-4, LMH cells, TMT, Notch signaling, inflammatory and immune response

## Abstract

Fowl adenovirus serotype 4 (FAdV-4) is recognized as a pathogen that causes hydropericardium syndrome. Irrespective of the pathway used by the virus to invade the chicken, the pathological characteristics of the disease include degeneration and necrosis of hepatocytes, formation of intranuclear inclusions, as well as inflammatory cell infiltration. Liver dysfunction constitutes one of the critical factors leading to death. Therefore, it is vital to investigate the virus-mediated severe pathological liver damage to further understand the pathogenesis of FAdV-4. Here, proteomics, a tandem mass tag (TMT)-based approach to directly analyze protein expression, was used to determine the protein expression during FAdV-4 proliferation in leghorn male hepatoma (LMH) cells. We identified 177 differentially expressed proteins associated with various biological processes and pathways. The functional enrichment analysis revealed that FAdV-4 could downregulate some signaling pathways in LMH cells, including NOD-like receptor signaling, RIG-I-like receptor signaling, NF-κB signaling, TNF signaling pathway, and Notch signaling, FoxO signaling, PI3K-Akt signaling, and autophagy. The results of proteomics screening suggested an association between FAdV-4 infection and Notch signaling in LMH *in vitro*, indicating that Notch signaling regulated the expression of inflammatory cytokines and interferons but not viral replication in LMH cells. These data contributed to the understanding of the immunopathogenesis and inflammopathogenesis of FAdV-4 infection and also provided valuable information for the further analysis of the molecular mechanisms underlying viral pathogenesis.

## Introduction

Since June 2015, there has been a progressive increase in the incidence of hydropericardium syndrome (HPS), which is characterized by pericardial effusion and hepatitis in Chinese chicken farms with a high mortality rate (Zhao et al., 2015; Niu et al., 2016; Wang and Zhao, 2019). In recent years, studies have identified a sporadic pattern in HPS as well as an expansion in its host range in other birds, such as ducks (Chen et al., 2017; Yu et al., 2018), geese (Ivanics et al., 2010), and ostriches (Changjing et al., 2016), which increases the complexity of the prevention and control of this disease. Previous studies have shown that HPS caused by highly pathogenic fowl adenovirus serotype 4 (FAdV-4) is an acute infectious disease, which can cause degeneration or necrosis of the heart, liver, kidney, thymus and other immune organs. However, no matter how the virus invades the chicken, the pathological characteristics of the liver are degeneration and necrosis of hepatocytes, formation of intranuclear inclusions and inflammatory cell infiltration. Liver dysfunction is one of the critical factors leading to death (Li et al., 2016; Niu et al., 2018). Therefore, it is vital to investigate the virus-mediated severe pathological liver damage to further understand the pathogenesis of FAdV-4.

Proteomics was used to determine the protein expression during FAdV-4 proliferation to elucidate the molecular mechanisms underlying the pathogenesis of FAdV-4 infection (Hou et al., 2021). We compared the expression of host proteins in leghorn male hepatoma (LMH) cells infected with the FAdV-4 strain (SDDM-15) to deepen our understanding of the interactions between host factors and FAdV-4. In the current study, we used a tandem mass tag (TMT)-based proteomic approach to reveal the protein expression difference at 24 h post-infection (hpi). Peptide segments were compared and matched with differentially expressed proteins (DEPs) related to cell metabolism using the Gene Ontology (GO) and Kyoto Encyclopedia of Genes and Genomes (KEGG) databases. The study aimed to provide valuable information for further analysis of the molecular mechanisms underlying viral pathogenesis and the immune responses elicited upon FAdV-4 infection.

Notch is an evolutionarily conserved signaling pathway from vertebrates to insects, which plays a vital role in proliferation, cell differentiation, survival and tissue homeostasis. Unlike mammals (Bray, 2006), only two Notch receptors (Notch1, Notch2) and four ligands Delta-like(Dll)-1, Dll4, Jagged (Jag)-1, and Jag2 have been identified in the chicken species (Hayashi et al., 1996; Myat et al., 1996; Crowe et al., 1998). Notch signaling widely known to play a vital role in the development of lymphocytes, as well as in the regulation of T cells differentiation and its functioning (Radtke et al., 2010). Therefore, it could also regulate the production of inflammatory factors and interferon. Previous studies have extensively studied the effect of Notch signaling on inflammatory and immune responses in mouse or human models; however, its studies in the chickens are rare. Since the proteomics screening suggested an association between FAdV-4 infection and Notch signaling in LMH *in vitro*, we hypothesized that Notch signaling played a vital role in the inflammatory and immune response to FAdV-4 infection. Our study is the first to report the Notch signaling-mediated regulation of the inflammatory cytokine and interferon expression but not viral replication in LMH cells. These data contribute to understanding the immunopathogenesis and inflammopathogenesis of FAdV-4 infection.

## Materials and methods

### Cell culture, virus and reagent

LMH cell line (ATCC^®^ CRL-2117™) was cultured in Dulbecco’s modified Eagle’s medium (DMEM) supplemented with 10% fetal bovine serum (FBS; Gibco, San Diego, CA, United States), penicillin (100 U/ml), and streptomycin (100 μg/ml) at 37°C in a humidified atmosphere of 5% CO_2_. The virulent FAdV-4 strain SDDM-4/15 used in the study was isolated from clinical samples of dead chickens and characterized by our research group (Niu et al., 2016). A γ-secretase inhibitor (DAPT) were purchased from Sigma (St. Louis, MO, United States), and reconstituted in dimethyl sulfoxide and stored in −80°C. The primary antibodies used in the study were specific for GAPDH (Goodhere, AB-P-R001), Hes1 (Affinity Biosciences, DF7569), AMPK (Affinity Biosciences, AF6423).

### Sample preparation and analysis

The experiment was divided into two groups, including mock and FAdV-4 groups. LMH cells were seeded in the six-well culture plates (1 × 10^6^ cells/well) and infected with FAdV-4 at a multiplicity of infection (MOI) of 1 for 24 hpi, while mock-infected cells were treated with an equal volume of DMEM. Then the cells were harvested for proteomics analysis. The mass spectrometry experiment method was performed based on the standardized process involving the following steps:

- Protein extraction and peptide enzymatic hydrolysis.
- TMT labeling.
- High pH reversed-phase peptide fractionation kit separation and AKTA purifier 100 fractionation- LC–MS/MS analysis-protein identification and quantitative analysis.
- Bioinformatics analysis.

### Total RNA extraction and real-time PCR

In order to further verify the transcription level of molecules in related signal pathways, LMH cells were seeded in the six-well culture plates (1 × 10^6^ cells/well) and infected with FAdV-4 at a MOI = 1 for 6–48 hpi. Then the cells were collected at different time points for transcription level analysis. Total RNA was extracted using FastPure^®^ Cell/Tissue Total RNA Isolation Kit V2 (Vazyme, Nanjing, China). First-strand cDNA was synthesized from total RNA (1 μg) using the HiScript^®^ II 1st Strand cDNA Synthesis Kit (Vazyme). All products were stored at −20°C prior to further use. Real-time (RT)-PCR oligonucleotide primers were designed using Primer 6.0 software,^1^ based on the published GenBank sequences (Table 1). qPCR was performed using the Taq Pro Universal SYBR qPCR Master Mix (Vazyme) and the ABI StepOne System (Applied Biosystems, Foster City, CA, United States). The qPCR was conducted using a total volume of 20 μl, with the following amplification steps: 95°C for 30 s, followed by 40 cycles of denaturation at 95°C for 10 s, and extension at 60°C for 30 s, followed by dissociation curve analysis. Each sample was analyzed in triplicates. Data were calculated based on the 2^−ΔΔCt^ method. The relative mRNA expression was normalized to that of *β*-actin.

**Table 1:** The primers used in the present study.

Gene symbol	Primers sequence	Products size (BP)
*Β-ACTIN*	F: 5′- CCAGCCATGTATGTAGCCATCCAG-3′ R: 5′- GGTAACACCATCACCAGAGTCCATC-3′	93
*Β-ACTIN*	F: 5′- CCAGCCATGTATGTAGCCATCCAG-3′ R: 5′- GGTAACACCATCACCAGAGTCCATC-3′	93
*NOTCH 1*	F: 5′- CTGTGAGTGCGTGGCTGGTTATC-3′ R: 5′- CATTCTGGCATGGGTGGGACAAG-3′	80
*NOTCH 2*	F: 5′- GAGTGGATGAACCGCATGGAGATG-3′ R: 5′- AGGTGCTTCGTGTCTGCTTGAAC-3′	131
*JAGGED1*	F: 5′- AATGGAGGAACTTGCCGAGACTTG-3′ R: 5′- CATCACACTGGCTGTCACGAGAG-3′	100
*JAGGED2*	F: 5′- CTGCTACTGCTGCTGCTCTTGG-3′ R: 5′- TGACATTCCGCACCGAGTTAAGC-3′	91
*DLL1*	F: 5′- CAACGAATGTGATGCCAACCCTTG-3′ R: 5′- CCTCCATTGAAGCACGGTCCATC-3′	150
*DLL4*	F: 5′- GGGCTGGTGGCTTTGCTCATAC-3′ R: 5′- GGTTGTTCATCGTCTCCAAGTCCTG-3′	97
*HES1*	F: 5′- AGCGAGTGCATGAACGAAGTGAC-3′ R: 5′- GGGTAGTTGATGGCGTTGATCTGG-3′	125
*HES5*	F: 5′- CAGAGACACCAACCCAACTCCAAG-3′ R: 5′- CTTCTTTGAGGCACCAGGCATACC-3′	148
*HEY1*	F: 5′- CGCCTTTGGACATCACCCTCAC-3′ R: 5′- AGAAGATGCTGTGGTGCTGGTATTG-3′	85
*PTEN*	F: 5′- CCCTTTGAAGACCATAACCCACCAC-3′ R: 5′- TTCGTCCCTTTCCAGCTTTACAGTG-3′	127
PIK3CA	F: 5′- AGTGGCTCAAGGACAAGAACAAAGG-3′ R: 5′- GCCCAGTATAAAGGTAGCGACACAG-3′	101
*AKT1*	F: 5′- TCACTCCTCCTGACCAAGATGACAG-3′ R: 5′- GCGGTTCCACTGGCTGAATAGG-3′	94
*FOXO3*	F: 5′- TCAAGGACAAGGGCGACAACAAC-3′ R: 5′- GCTCTTCCCAGTGCCTTCGTTC-3′	113
*P27*	F: 5′- GAAGGCAGGTACGAGTGGCAAG-3′ R: 5′- GGTTTGGCAATTCCCGTTTACATCC-3′	135
*P53*	F: 5′- GCGGAGGAGATGGAACCATTGC-3′ R: 5′- GCTCCTGCCAGTTGCTGTGATC-3′	118
TSC1	F: 5′- AGCAGTAAAGGTGGCAGCAACAG-3′ R: 5′- ACAGTCAGTGGGACAGTAGTGGAG-3′	122
MTOR	F: 5′- AGCCTGCCTTATCCTCACCACTC-3′ R: 5′- TGGATTCGGTCATCACGGTTCATTC-3′	144
ULK1	F: 5′- GAGCAAGAGCACACCGACATCC-3′ R: 5′- TTTCAGGGCAGCAATCTCCATCAC-3′	84
AMPK	F: 5′- ACCATCTGTCTCGCCCTCATCC-3′ R: 5′- AATGCCACTTCGCTCTTCTTACACC-3′	132
PIK3C3	F: 5′- GCTGAATAAGGAGATGGTGGAAGGC-3′ R: 5′- AGTTGGAGTACCTGCGGAGATGG-3′	110
LAMP1	F: 5′- GAGACAAGTTTGGGGCAGTGGAAG-3′ R: 5′- GCGATCAGGACAATCAGAACCAGAC-3′	109
*MDA5*	F: 5′- GTGGCTTCAAGTGGCTCAGGAG-3′ R: 5′- AATACTCTTCTGGCGGCATCTCTTG-3′	112
*TLR21*	F: 5′- TCTCACAGGCGGAGGTCTTCAC-3′ R: 5′- GCGAGGTTGGATGTCAGAGATGTC-3′	116
*NOD1*	F: 5′- CTGGTGCTGCTGGTAAGCCTTC-3′ R: 5′- CTCAGTTCCTGTCCGTGCTGTAAG-3′	81
*NLRP3*	F: 5′- GCTCCTTGCGTGCTCTAAGACC-3′ R: 5′- TTGTGCTTCCAGATGCCGTCAG-3′	150
*NF-ΚB(P50)*	F: 5′- GGTGGTATGTGGGAAGGCTTTGG-3′ R: 5′- CAGATGCTGGCTTTGTGATGTTGAC-3′	115
*IL6*	F: 5′- GAAATCCCTCCTCGCCAATCTGAAG-3′ R: 5′- GCCCTCACGGTCTTCTCCATAAAC-3′	108
*TNFΑ*	F: 5′- CTCAGGACAGCCTATGCCAACAAG-3′ R: 5′- GGCGGTCATAGAACAGCACTACG-3′	86
*CGAS*	F: 5′- AAACACCTGGCGACTCTCTTTCTC-3′ R: 5′- TTCCTGCAACACTTCACTCCATCG-3′	105
*TBK1*	F: 5′- TGGAATACAGAAGGCAGCAAACGG-3′ R: 5′- GCAAGGACAGGTGTGAGCAGTAC-3′	105
*STING*	F: 5′- AGCTACTGGTCCTTGCCCTTGG-3′ R: 5′- CCGTGAGCGACATTCTTCTTGGAG-3′	91
*IFNΑ*	F: 5′- TCCTTCAGAATACGGCTCCACCTC-3′ R: 5′- TGGCTGCTTGCTTCTTGTCCTTG-3′	90
*IFNΒ*	F: 5′- TCCTTCAGAATACGGCTCCACCTC-3′ R: 5′- TGGCTGCTTGCTTCTTGTCCTTG-3′	108

### Notch signaling pathway inhibition assay

To further study the role of Notch signaling, LMH were pretreated with DAPT (10 μM) for 1 h, and then infected with FAdV-4 (MOI 1) for 48 h with DAPT (10 μM). Cell samples were harvested to determine the other related protein transcription level, and viral titers by TCID_50_ assay.

### Statistical analysis

All data were expressed as means ± standard deviation (SD). Significance was determined with the two-tailed independent Student’s *t*-test. The probability (*P*) value less than 0.05 was considered statistically significant.

## Results

### Analysis of proteomics based on LC–MS/MS

**Figure 1:**
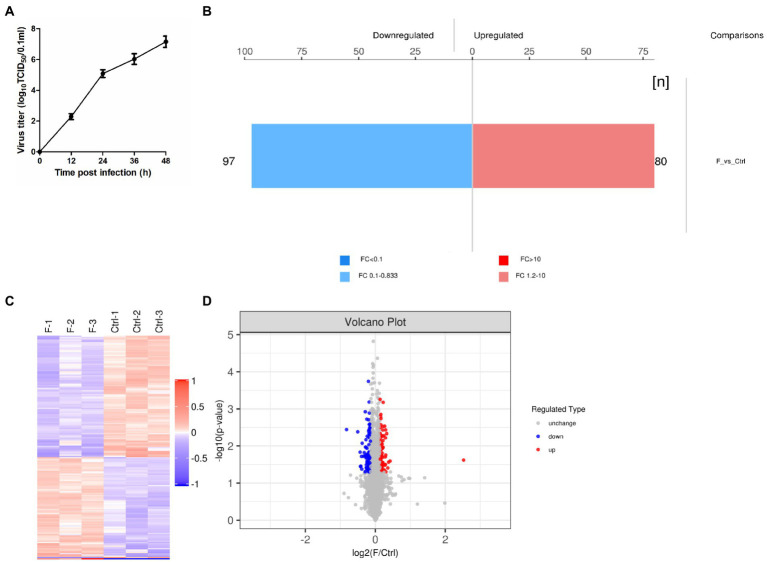
FAdV-4 induction of differentially expressed proteins (DEPs) in LMH cells at 24 hpi (*p* < 0.05, |log_2_(fold-change)| > 1.2). **(A)** One-step growth curve of FAdV-4 in LMH cells. **(B)** Histogram of protein quantitative difference results. **(C)** The heatmap shows the protein expression patterns, and the volcano plot displays the number of DEPs. **(D)** Volcano plots of DEPs between control and FAdV-4 groups. *X*-axis: base-2 logarithm of fold-change (experimental group/control group); y-axis: negative logarithm of *p*-value.

Based on the results of previous studies (Ma and Niu, 2022) and the growth curve of the FAdV-4 (Figure 1A), the optimal time point of proteomic analysis was set at 24 hpi after FAdV-4 infecting LMH cells. At this time point, the virus titer approximately 10^5.1^ TCID_50_/0.1 ml was a period of rapid proliferation, and half of the cells had been infected, so the state of cells might change. Therefore, screening of DEPs might have a serious effect on the later stage. In this study, TMT label-enrichment method was used, followed by LC–MS/MS analysis to comprehensively investigate the interaction between host proteins and FAdV-4 infection in LMH cells. We identified 6,169 proteins by mapping the obtained peptides against the reference database for the chicken species. Statistically significant DEPs were detected using the *t*-test (*p* < 0.05). The abundances of the proteins were filtered using the thresholds of *p* < 0.05 and |log_2_(fold-change)| > 1.2. The proteome profiles of infected cells and mock control cells were compared to determine the effects of FAdV-4 infection in LMH cells. Thus, 177 DEPs (97 upregulated, 80 downregulated) were identified in LMH cells after FAdV-4 infection using these standards. These results were visualized by clustering the samples according to differential treatment and by constructing a volcano plot of the DEPs (Figures 1B–D). The DEPs are listed in Supplementary Table S1.

### Functional analysis of identified cellular proteins

**Figure 2:**
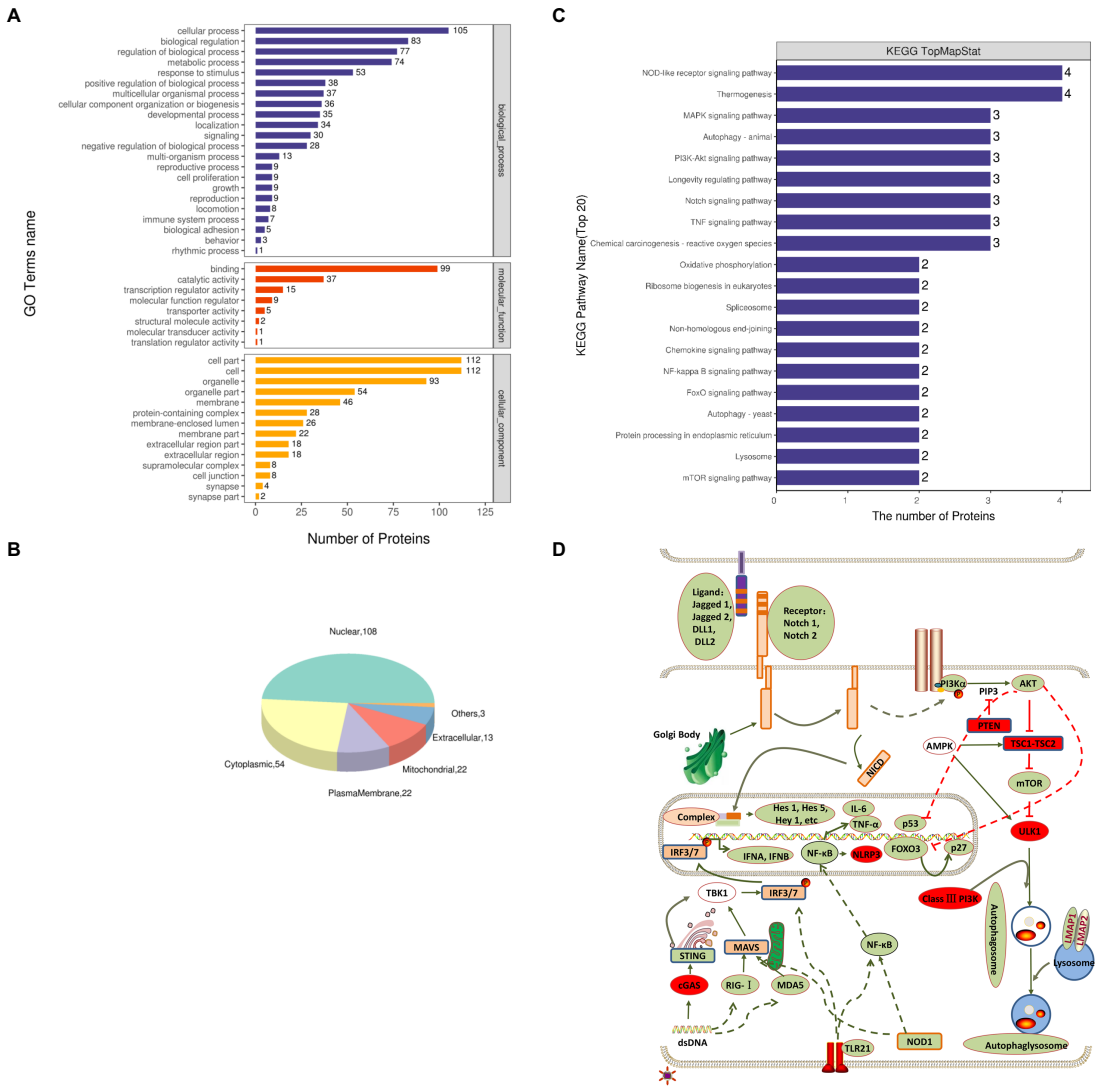
Gene Ontology (GO) and Kyoto Encyclopedia of Genes and Genomes (KEGG) enrichment analyses of DEPs. **(A)** Total GO categories in biological process, molecular function, and cellular component at 24 hpi. **(B)** Top 20 KEGG pathways for differentially expressed proteins. **(C)** Subcellular localization pie chart of differentially expressed proteins. **(D)** Schematic diagram of signal pathway changes in LMH cells infected with FAdV-4. The transcriptome levels of molecules that significantly increased at 24 hpi were filled with red, and those that significantly decreased were filled with green.

The functional significance of these identified proteins was analyzed using the GO database. According to the GO functions, the DEPs were classified into the biological process (BP), molecular function (MF), and cellular component (CC). The GO analysis showed that in BP classification, cellular process, biological regulation, regulation of the biological process, and metabolic process were the major functional classes (>35% for each class) of the identified proteins; in MF classification, DNA binding (>55%) was the major functional class of the identified proteins; and in CC classification, cell part, cell, and organelle (>52%) formed the major class for the identified proteins (Figure 2A). Subcellular localization of all DEPs was analyzed using the subcellular structure prediction software CELLO. The results showed that the number of DEPs was 108 in the nucleus, 54 in the cytoplasm, 22 in the plasma membrane, 22 in mitochondria, 13 in extracellular, and three in others (Figure 2B). The DEPs enriched in the significant categories at 24 hpi are listed in Supplementary Table S2. The number of DEPs was counted, and the top 20 pathways with the largest number of DEPs were presented in the form of a histogram (Figure 2C). Among these pathways, the NOD-like receptor signaling, RIG-I-like receptor signaling, NF-κB signaling, and TNF signaling pathway were the pathways associated with innate immune response and inflammatory response; Notch signaling, FoxO signaling, PI3K-Akt signaling pathway, autophagy were pathways associated with cell differentiation, apoptosis, proliferation, autophagy. Next, we further screened these pathways to identify any connections (Figure 2D).

### Verification of pathways through RT-PCR

**Figure 3:**
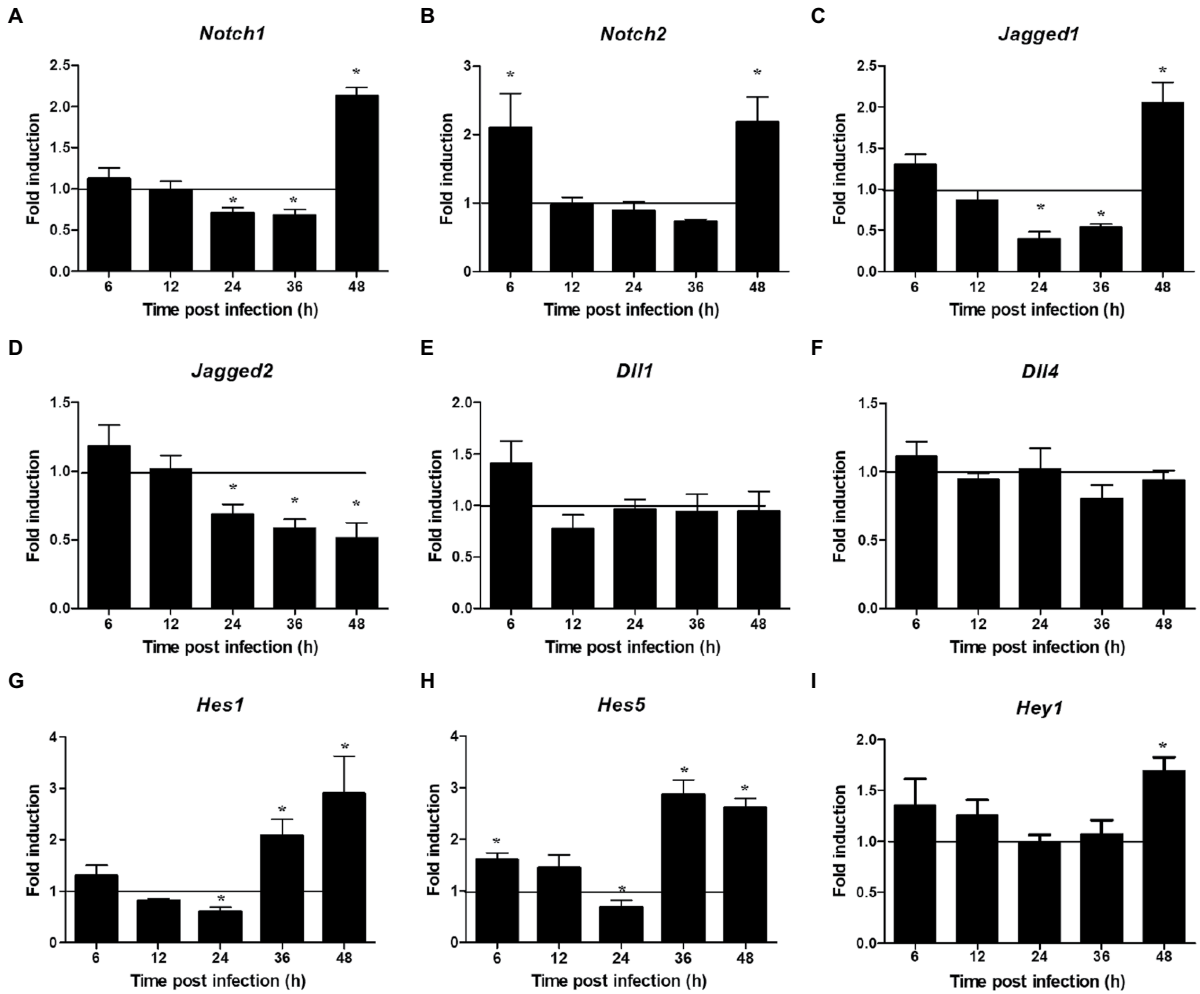
Expression profile of Notch molecules in LMH during FAdV-4 infections at 6–48 hpi. **(A)** *Notch 1*, **(B)** *notch 2*, **(C)** *jagged 1*, **(D)** *jagged 2*, **(E)** *dll 1*, **(F)** *dll 4*, **(G)** *has 1*, **(H)** *has 5*, **(I)** *hey 1*. Relative expression is normalized to *β-actin*. The y-axis represents the fold change in target gene expression in LMH compared with that of the control and the above line (*y* = 1) on the *x*-axis represents the control. Data are shown as mean [standard deviation (SD)] with *n* = 3 per group. ^*^p < 0.05.

**Figure 4:**
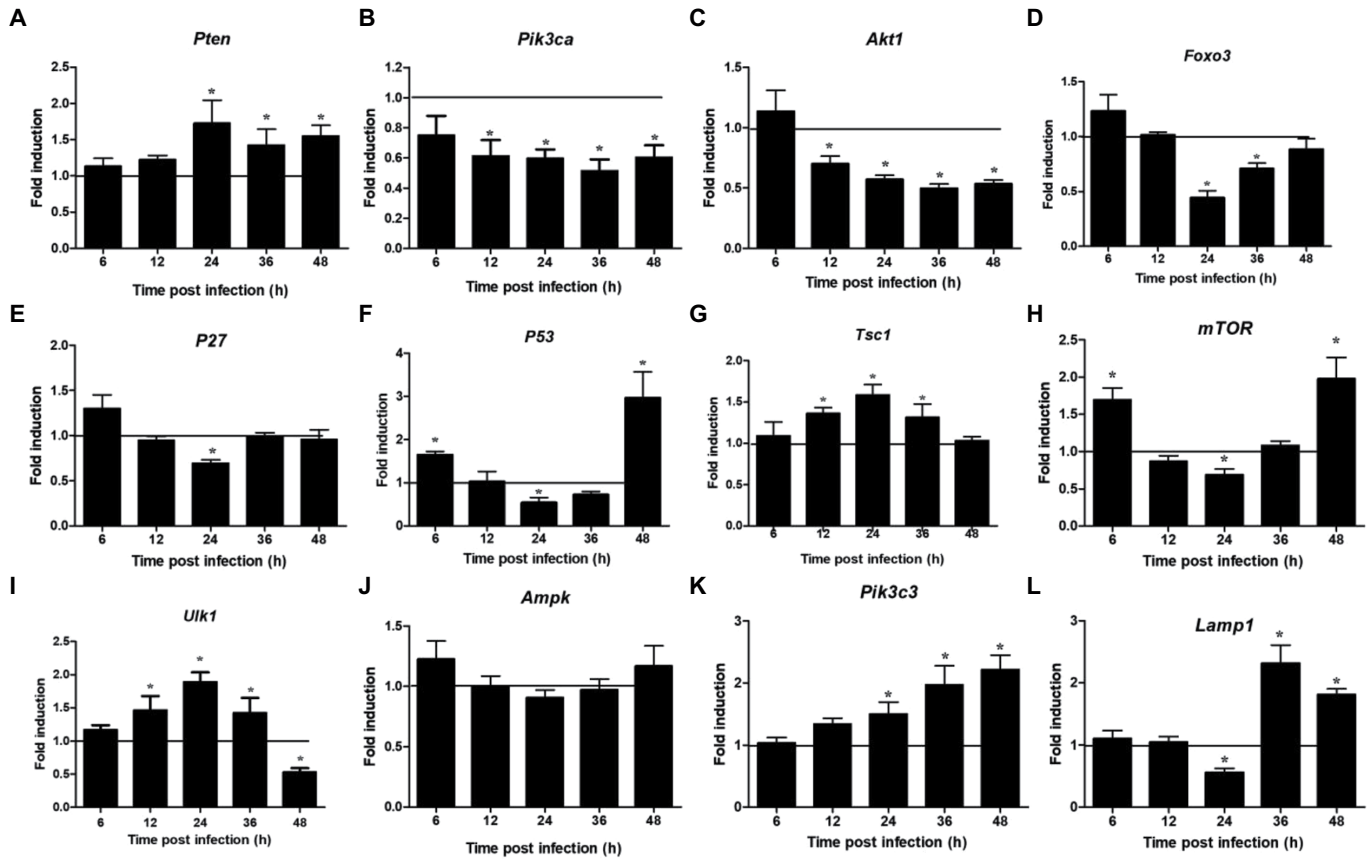
Expression of downstream Notch signaling pathway and autophagy related factors in LMH cells infected with FAdV-4 at 24–48 hpi. **(A)** *Pten*, **(B)** *pik3ca*, **(C)** *akt 1*, **(D)** *foxo 3*, **(E)** *p27*, **(F)** *p53*, **(G)** *tsc 1*, **(H)** *mTOR*, **(I)** *ulk 1*, **(J)** *ampk,* **(K)** *pik3c3*, **(L)** *lamp 1*. Relative expression is normalized to *β-actin*. The y-axis represents the fold change in target gene expression in FAdV-4 group compared with that of the control and the above line (*y* = 1) on the *x*-axis represents the control. Data are shown as mean (SD) with *n* = 3 per group. ^*^p < 0.05.

The proteins related to the signaling pathway were selected for verification through RT-PCR to confirm the effect of the selected signal pathway. First, we measured the expression of Notch molecules, including Notch receptors (1, 2), ligands (Dll1, 4, and jagged1, 2), and the representative target *hes1*, *hes5*, and *hey1* in LMH cells after FAdV-4 infection. During FAdV-4 infection, the expressions of *notch1*, *jagged1*, and *jagged2* were decreased compared with the mock-infected group at 24–36 hpi (Figure 3). Consistent with the decreased expressions of Notch receptor and the ligands, the expressions of target genes *hes1* and *hes5* were restrained at 24 hpi, suggesting that FAdV-4 infection inhibited the activation of Notch signaling in LMH at 24 hpi. In FAdV-4 group, *Notch1*, *notch2, jagged1*, *hes1*, *hes5*, and *hey1* showed similar significantly increasing trends at 48 hpi, compared with the control group. Moreover, *dll1* and *dll4* mRNA expression were no significant change compared with the control group during 6–48 hpi. PI3K-Akt signaling pathway widely exists in cells and is a signal transduction pathway involved in the regulation of cell growth, proliferation, and differentiation. The Notch signal can regulate the activity of the PI3K-Akt signaling pathway. Next, we detected the expression of genes related to the P13K–Akt signaling pathway. The results of RT-PCR showed that the expression of *pik3ca* and *aktl* decreased significantly at 24–48 hpi (Figures 4B,C); the expression of *pten*, one of the inhibitors of the PI3K-Akt signaling pathway, was significantly increased at 24–48 hpi (Figure 4A). On the contrary, the expression of *foxo3* (Figure 4D) and *p53* (Figure 4F), the negative downstream regulator of the P13K–Akt signaling pathway, decreased significantly at 24 hpi, along with the expression of the *foxo3* downstream effector gene *p27* (Figure 4E). However, *p*53 levels were significantly increased at 48 hpi (Figure 4F). In addition, genes related to the autophagy pathway downstream of the PI3K-Akt signaling pathway were also analyzed. The mRNA expression of *Tsc1* was significantly elevated at 12–36 hpi (Figure 4G), the expression of *mTOR* was only significantly decreased at 24 hpi, but *mTOR*s mRNA expression was significantly enhanced at 48 h (Figure 4H), and the expression of *ulk1* was also significantly elevated during 12–36 hpi (Figure 4I). Additionally, the expression of *ampk* did not change significantly during 6–48 hpi (Figure 4J), and that of III *pi3k* (*pik3c3*) was significantly increased at 24–48 hpi (Figure 4K). The expression of lysosome-related genes (*lamp1*) was also found to have decreased significantly at 24 hpi, and then increased significantly at 36–48 hpi (Figure 4L).

**Figure 5:**
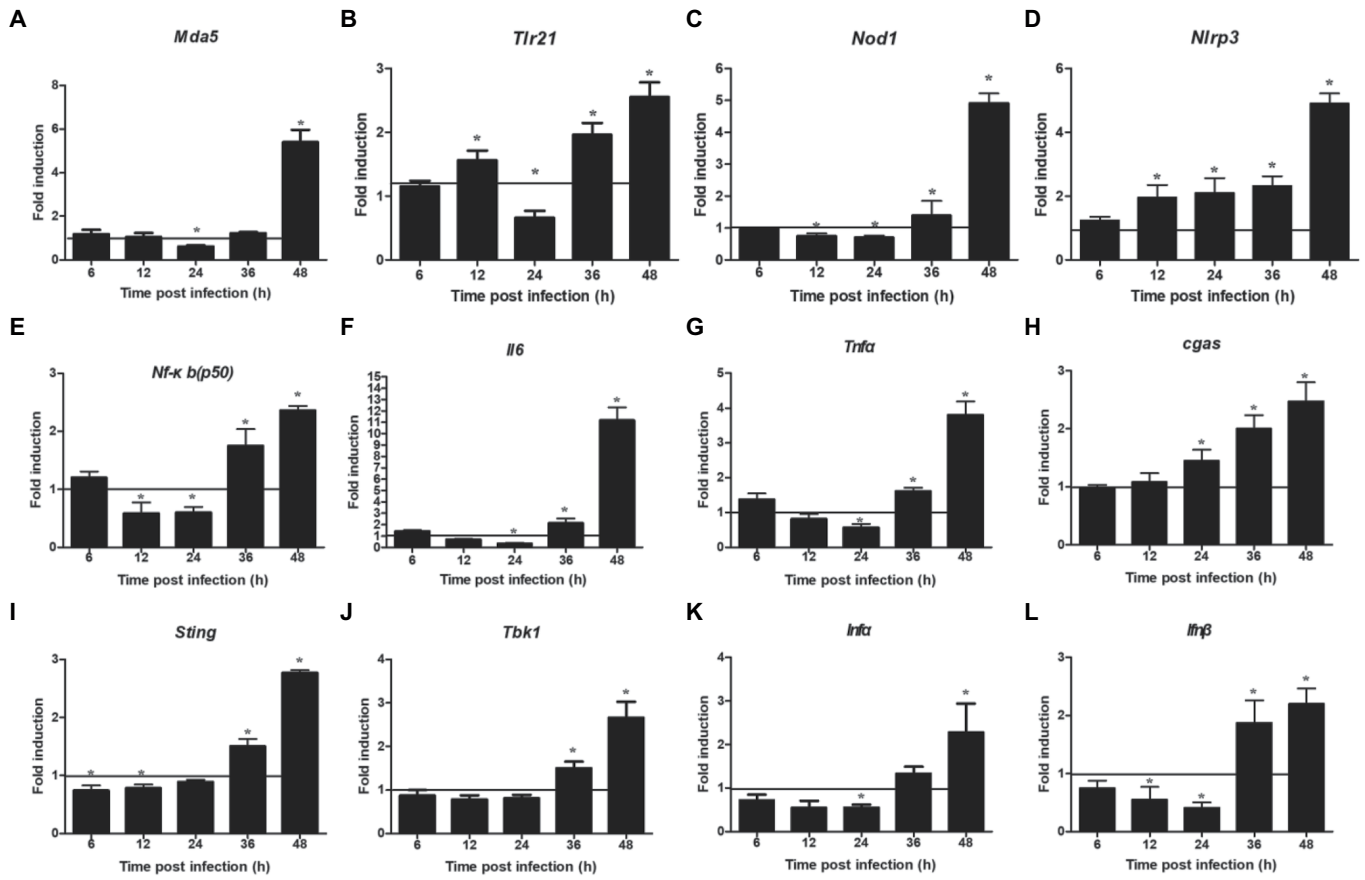
Expression levels of inflammatory and immune related factors. **(A)** *Mda 5*, **(B)** *trl 21*, **(C)** *nod 1*, **(D)** *nlrp 3*, **(E)** *nf-κb(p50)*, **(F)** *il 6*, **(G)** *tnf α*, **(H)** *cgas*, **(I)** *tbk 1*, **(J)** *sting,* **(K)** *ifn α*, **(L)** *ifn β*. Relative expression is normalized to *β-actin*. Fold induction as compared to mock-infected samples, the solid line is representative of 1 fold for mock samples. Data are shown as mean (SD) with *n* = 3 per group. ^*^p < 0.05.

NOD-like receptor signaling pathway showed a downregulated expression. Next, we detected the expression of two related inflammasome, and the results showed that the expression of *nod1* decreased significantly at 12–24 hpi and enhanced significantly at 36–48 hpi (Figure 5C), but that of *nlrp3* increased significantly at 12–48 hpi (Figure 5D). In addition, we also detected the expression of two other DNA virus receptors (MDA5 and TLR21). The results showed that their expression was also significantly downregulated at 24 hpi, but significantly upregulated at 48 hpi (Figures 5A,B). Furthermore, the mRNA expressions of *nf-κb (p50)*, *il6*, and *tnfα* were downregulated at 24 hpi and upregulated at 36–48 hpi (Figures 5E–G). Regarding the innate immune response, the mRNA expressions of *ifnα* and *ifnβ* were significantly downregulated at 24 hpi and upregulated at 36–48 hpi (Figures 5K,L), but that of *cgas* was increased significantly at 24–48 hpi (Figure 5H). The mRNA expressions of *sting* and *tbk1* also decreased compared with the control group, but there was no significant difference at 24 hpi, and then increased significantly at 36–48 hpi (Figures 5I,J).

### Effect of Notch signaling on inflammatory cytokine expression in LMH during FAdV-4 infection

**Figure 6:**
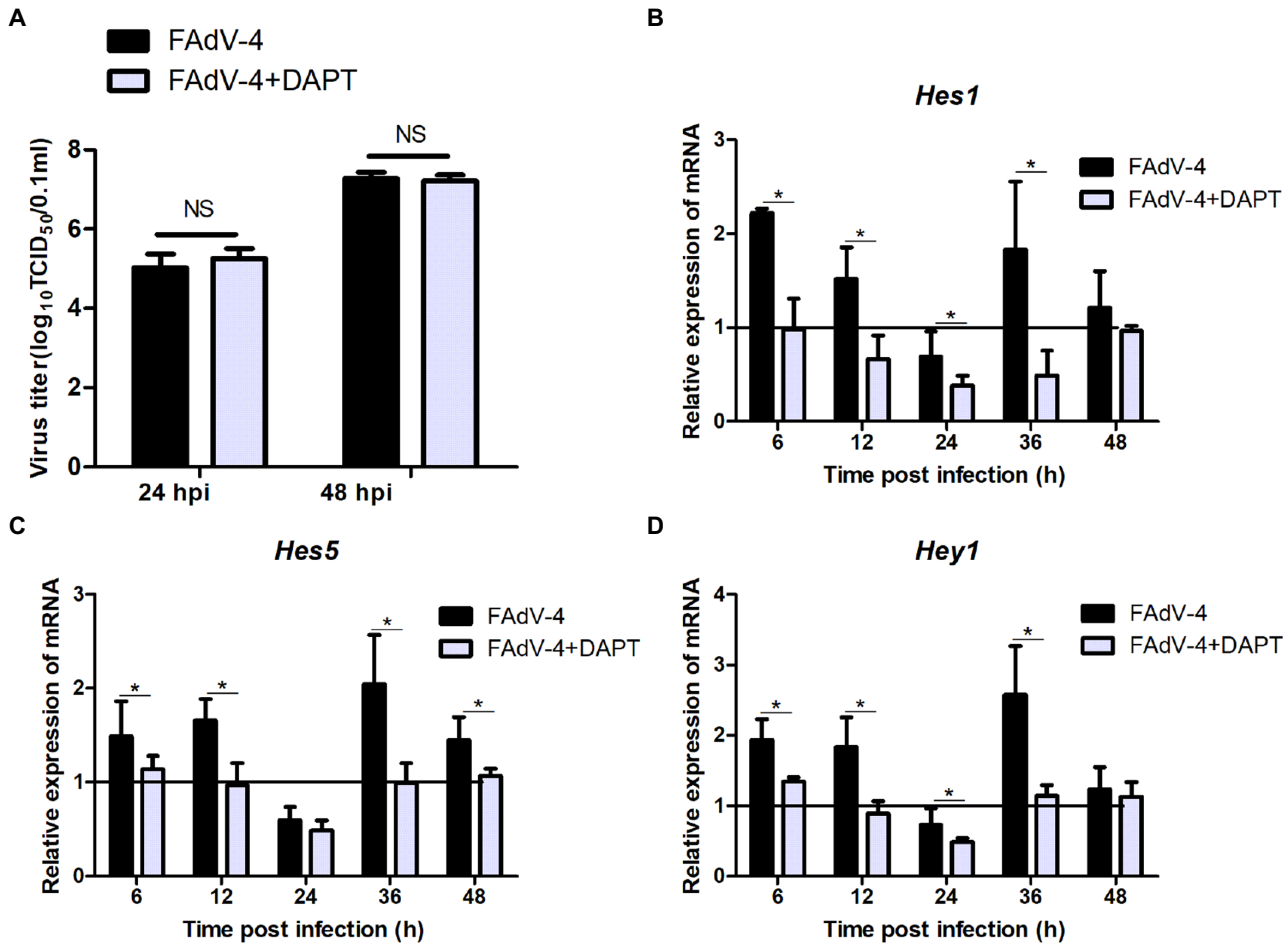
Effect of Notch signaling on viral replication during FAdV-4 infection in LMH. **(A)** Supernatants were harvested and tittered by TCID_50_ assay at 24 and 48 hpi. **(B–D)** Cells were harvested, and then the expression of the target gene *hes1*, *hes5*, and *hey1* were evaluated by qPCR. The y axis represents the fold change in target gene expression in FAdV-4 group and FAdV-4+ DAPT group compared with that of the control and the above line (*y* = 1) on the *x*-axis represents the control. Data are shown as mean (SD) with *n* = 3 per group. NS, not statistically significant; ^*^p < 0.05, represents the comparison between group FAdV-4 and group FAdV-4+ DAPT.

**Figure 7:**
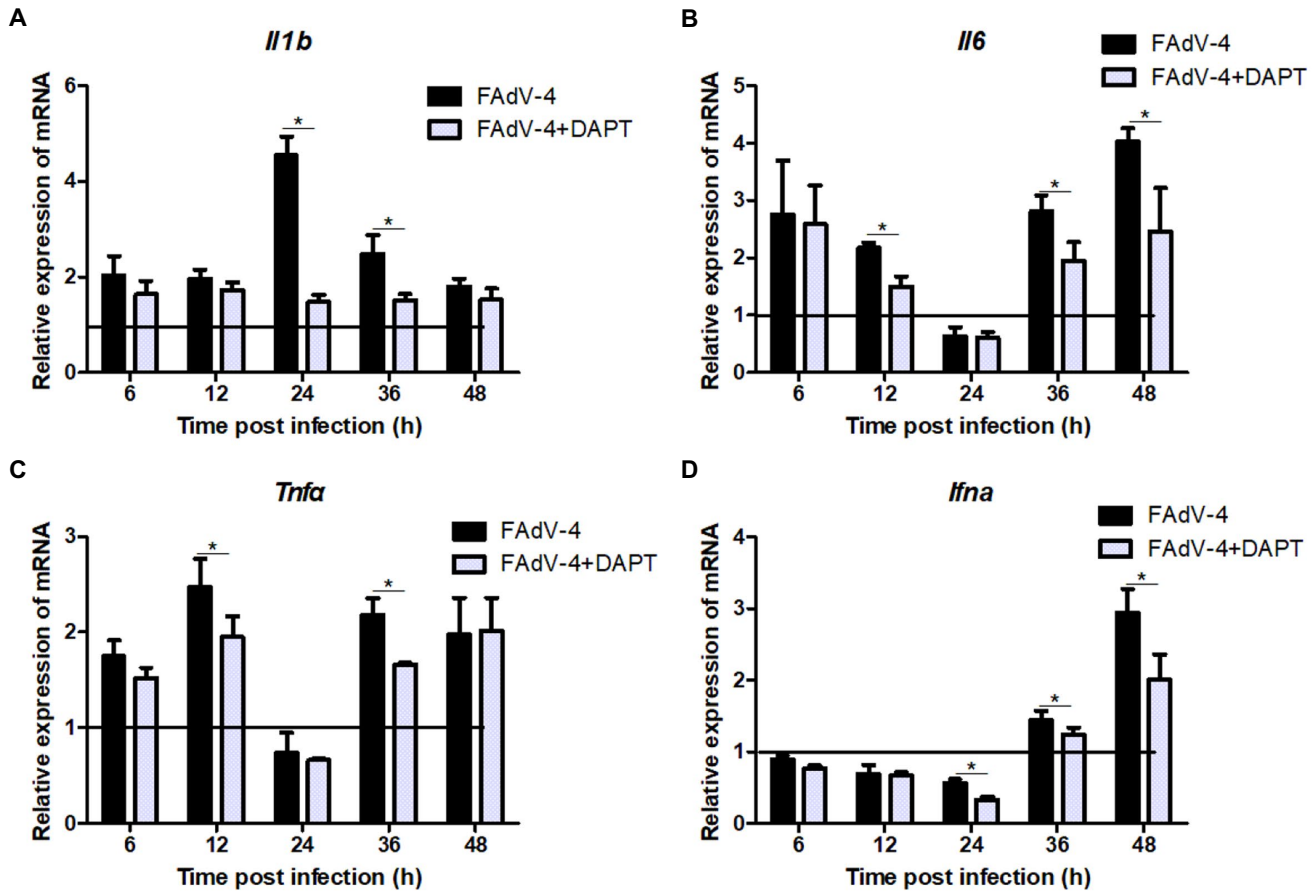
Restraining Notch signaling with DAPT inhibits expression of the inflammatory and immune cytokines induced by FAdV-4 infection. Cells were harvested at different time, then expression of the target gene *il1b* **(A)**, *il6* **(B)**, *tnfα* **(C)** and *ifnα* **(D)** was evaluated by qPCR. The y axis represents the fold change in target gene expression in in FAdV-4 group and FAdV-4+ DAPT group compared with that of the control and the above line (*y* = 1) on the *x*-axis represents the control. Data are shown as mean (SD) with *n* = 3 per group. ^*^p < 0.05, represents the comparison between group FAdV-4 and group FAdV-4+ DAPT.

The results of the proteomic analysis showed that the Notch signaling pathway was significantly downregulated at 24 hpi (Figure 2C) and was also related to other signal pathways (Figure 2D). We first revealed the effect of Notch signaling on FAdV-4 replication in LMH to understand the functional role of Notch signaling during virus infection. We treated LMH with DAPT to suppress Notch signaling and compared the viral replication by TCID_50_ and *hes1*, *hes5*, and *hey1* expression by qPCR between FAdV-4 and DAPT-treated groups during FAdV-4 infection. There was no difference in the viral replication between FAdV-4 and DAPT-treated groups at 24 and 48 hpi (Figure 6A). However, the blockade of activation of Notch signaling by DAPT treatment during FAdV-4 infection, which significantly inhibited the expression of the target gene *hes1*, *hes5*, and *hey1* in comparison to the FAdV-4 group during 6–48 hpi (Figures 6B–D). Studies have shown that Notch signaling helps regulate the expression of inflammatory cytokines and immune responses under viral infection (Mukherjee et al., 2019) and proinflammatory stimuli (TLR ligands, inflammatory cytokines, etc.) (Fung et al., 2007; Hu et al., 2008; Foldi et al., 2010; Tsao et al., 2011). Therefore, we investigated the role of Notch signaling in the inflammatory cytokine and immune response expression in LMH cells during FAdV-4 infection. Compared with the FAdV-4 group, DAPT treatment significantly reduced the mRNA expressions of the inflammatory and immune cytokines, *il1b*, *tnfα*, *il6*, and *ifnα*, which were induced in LMH cells during FAdV-4 infection (Figure 7). In FAdV-4 + DAPT group, *il1b* mRNA level showed significantly decreasing trends at 24–36 hpi, compared with the FAdV-4 group (Figure 7). And *tnfα*, *il6*, and *ifnα* mRNA expression, were significantly inhibited at different time points by DAPT treatment during FAdV-4 infection. Therefore, these results revealed that Notch signaling helps regulate the inflammatory and immune response of LMH cells to FAdV-4 infection.

## Discussion

All viruses are obligate parasites and undergo evolutionarily conserved life cycles that basically depend on virus-host interactions, frequently mediated by protein–protein associations. When the virus enters the host, it tries to hijack and manipulate the host cell to ensure its own survival and replication (Lum and Cristea, 2016; Coombs, 2020). The last decade has witnessed the increasing application of proteomics to virology studies (Cristea and Graham, 2015). Because of the high morbidity and mortality of HPS, it is important to use proteomics methods to further understand the FAdV-4 infection and pathogenesis. This study employed a TMT-based approach to analyze changes in the proteins whose expression was differentially regulated in LMH cells during FAdV-4 infection. Our previous research results had shown that the host cells changed significantly at 24 hpi (Niu et al., 2018; Ma and Niu, 2022), and the protein level also changed accordingly. Therefore, we performed proteomic analysis of LMH cells 24 h after virus infection. Through analysis, we identified 177 DEPs (97 upregulated, 80 downregulated) in LMH cells after FAdV-4 infection 24 hpi (Figure 1). These DEPs were mainly involved in cellular processes, biological regulation, regulation of the biological process, and metabolic process (Figure 2A). Notch is an evolutionarily conserved signaling pathway from vertebrates to insects, which plays a vital role in proliferation, cell differentiation, survival and tissue homeostasis (Falo-Sanjuan and Bray, 2020). In addition, Notch signaling known to play a vital role in the development of lymphocytes, as well as in the regulation of T cells differentiation and its functioning (Maillard et al., 2005; Tanigaki and Honjo, 2007; Radtke et al., 2010). According to our previous findings, FAdV-4 caused immunosuppression, leading to the loss of immune cells in immune organs (Niu et al., 2019). Thus, whether this signaling pathway participates in the regulation of immune-inflammatory response would be our next focus. The top 20 KEGG pathways were selected during FAdV-4 infection in LMH cells including the Notch pathway.

This research provides novel information regarding the expression profile of Notch molecules and the effects of Notch signaling on FAdV-4 infection in LMH cells. We proved that FAdV-4 infection inhibit expression of the target genes *hes1* and *hes5*, consistent with restrain of Notch signaling which is associated with Notch ligands including *jagged1* and *jagged2* as well as the Notch receptor 1, but interestingly, not the Notch receptor 2 at 24 hpi. Although Notch1 and Notch2 receptors are expressed in LMH cells, the different roles of the two receptors during FAdV-4 infection remain to be investigated. Other studies have shown that the expression of the two Notch receptors is usually closely related to cell fate, and Notch receptor 2 is more related to proliferation (Vázquez-Ulloa et al., 2018; Jakovljevic et al., 2020; Giovannini et al., 2021). This may explain the reason why FAdV-4 infection has less impact on Notch receptor 2. In the early stage of FAdV-4 infection, the amount of virus was small and the proliferation of cells was not affected, so Notch receptor 2 had not been significantly affected. With lastingness of infection, the expression of target gene *hes1*, *hes5* and *hey1* significantly increased, consistent with activation of *jagged1* and Notch receptors (1 and 2). Collectively, the level of Notch pathway related genes and proteins was downregulated at 24 hpi, but the transcription level was significantly upregulated at 36–48 hpi (Figure 3). Notch signaling pathway is involved in cell proliferation and differentiation, when its expression was blocked, which will further affect the life activities of cells (Artavanis-Tsakonas et al., 1999). This may partly explain why the virus infects the host, which slowed down proliferation and hindered growth. In addition, we detected the transcription of some pattern receptors that recognize DNA viruses and the transcription level of interferon. Consistent with the expected results, their transcription level also decreased significantly. Aside from natural antiviral effect weakened, the inflammatory response that induced by FAdV-4 at 24 hpi decreased significantly (Figure 5). We further establish that the effects of Notch signaling on the regulation of inflammatory and immune response but not viral replication in LMH during FAdV-4 infection. By targeting signaling molecule, we show that blockade of γ-secretase by DAPT inhibits transcription of IL-1β, TNF-α, IL-6, and IFN-α, but not viral replication. In aggregate, our data identify Notch as an important signaling pathway that could contribute to the regulation of inflammatory and immune response induced by FAdV-4 infection, but the specific mediating mechanism is unclear. Inflammatory and immune response are important for protecting the host against infections. However, dysregulated inflammatory cytokines or interferons may cause pathological reactions in the host (Vázquez-Ulloa et al., 2018). Therefore, we speculated that FAdV-4 may inhibit the excessive inflammatory and immune response of the host through Notch signaling pathway in the early stage of infection, which will assist in virus replication in the host. Notch signaling also plays a crucial regulatory role in the intensified inflammatory response of host cells 36–48 h after viral infection.

The Notch pathway is one of the most frequently activated signaling pathways in human liver diseases. Since Notch signaling regulates multiple cellular activities, the dysregulation of the Notch cascade contributes to various pathological processes (Kopan and Ilagan, 2009). It has been found that the Notch signal is very critical for the regulation of autophagy. Furthermore, the PI3K-Akt signaling pathway plays a vital role in regulating autophagy, and its downstream effectors, such as p53 and p27, also play a decisive role in the fate of cells (Wang and Zhang, 2019; Zhang and Zhang, 2019). Combined with the results of proteomic analysis, we found that the PI3K-Akt, FoxO, and autophagy signaling pathways were downregulated. Therefore, we used RT-PCR to detect the expression of the related genes and found that they were indeed significantly reduced. Thus, these results suggested that Notch signaling participated in regulating genes critical for these signaling pathways, but specific key factors need further validation.

Thus, this is the first study to demonstrate that Notch, an important signaling pathway, could contribute to the regulation of inflammatory and immune response induced FAdV-4 infection in LMH cells using a TMT-based proteomic approach. Moreover, the significantly different proteins and signaling pathways identified would also aid in understanding the poorly understood molecular pathogenesis of FAdV-4.

The raw data of proteomics in this article can be obtained through the following website: https://www.iprox.cn/page/PSV023.html;?url=1659787855810agYv.

## Data availability statement

The datasets presented in this study can be found in online repositories. The names of the repository/repositories and accession number(s) can be found at: https://www.iprox.cn/page/PSV023.html;?url=1659787855810agYv.

## Author contributions

YN performed the most experiments, analyzed data, and wrote the manuscript. KD and KC performed some qPCR experiments. ZL and MW cultured LMH cells. YD contributed to design of the study and revised the manuscript. All authors contributed to the article and approved the submitted version.

## Funding

This study was funded by National Natural Science Foundation of China (31902232).

## Conflict of interest

The authors declare that the research was conducted in the absence of any commercial or financial relationships that could be construed as a potential conflict of interest.

## Publisher’s note

All claims expressed in this article are solely those of the authors and do not necessarily represent those of their affiliated organizations, or those of the publisher, the editors and the reviewers. Any product that may be evaluated in this article, or claim that may be made by its manufacturer, is not guaranteed or endorsed by the publisher.

## Footnotes

^1^ http://bioinfo.ut.ee/primer3-0.4.0/
