# Supplementary material for: TMT-based quantitative proteomics analysis reveals the role of Notch signaling in FAdV-4-infected LMH cell
Source: Front Microbiol. 2022 Sep 15;13:988259. doi: 10.3389/fmicb.2022.988259 (PMC9520525; doi:10.3389/fmicb.2022.988259)
Supplement: Supplementary file 3 [file Table_4.DOCX]

**Table 1.** The primers used in the present study

| Gene symbol | Primers sequence | Products size (bp) |
| --- | --- | --- |
| *β-actin* | F: 5ʹ- CCAGCCATGTATGTAGCCATCCAG-3ʹ  R: 5ʹ- GGTAACACCATCACCAGAGTCCATC-3ʹ | 93 |
| *β-actin* | F: 5ʹ- CCAGCCATGTATGTAGCCATCCAG-3ʹ  R: 5ʹ- GGTAACACCATCACCAGAGTCCATC-3ʹ | 93 |
| *Notch 1* | F: 5ʹ- CTGTGAGTGCGTGGCTGGTTATC-3ʹ  R: 5ʹ- CATTCTGGCATGGGTGGGACAAG-3ʹ | 80 |
| *Notch 2* | F: 5ʹ- GAGTGGATGAACCGCATGGAGATG-3ʹ  R: 5ʹ- AGGTGCTTCGTGTCTGCTTGAAC-3ʹ | 131 |
| *Jagged1* | F: 5ʹ- AATGGAGGAACTTGCCGAGACTTG-3ʹ  R: 5ʹ- CATCACACTGGCTGTCACGAGAG-3ʹ | 100 |
| *Jagged2* | F: 5ʹ- CTGCTACTGCTGCTGCTCTTGG-3ʹ  R: 5ʹ- TGACATTCCGCACCGAGTTAAGC-3ʹ | 91 |
| *Dll1* | F: 5ʹ- CAACGAATGTGATGCCAACCCTTG-3ʹ  R: 5ʹ- CCTCCATTGAAGCACGGTCCATC-3ʹ | 150 |
| *Dll4* | F: 5ʹ- GGGCTGGTGGCTTTGCTCATAC-3ʹ  R: 5ʹ- GGTTGTTCATCGTCTCCAAGTCCTG-3ʹ | 97 |
| *Hes1* | F: 5ʹ- AGCGAGTGCATGAACGAAGTGAC-3ʹ  R: 5ʹ- GGGTAGTTGATGGCGTTGATCTGG-3ʹ | 125 |
| *Hes5* | F: 5ʹ- CAGAGACACCAACCCAACTCCAAG-3ʹ  R: 5ʹ- CTTCTTTGAGGCACCAGGCATACC-3ʹ | 148 |
| *Hey1* | F: 5ʹ- CGCCTTTGGACATCACCCTCAC-3ʹ  R: 5ʹ- AGAAGATGCTGTGGTGCTGGTATTG-3ʹ | 85 |
| *Pten* | F: 5ʹ- CCCTTTGAAGACCATAACCCACCAC-3ʹ  R: 5ʹ- TTCGTCCCTTTCCAGCTTTACAGTG-3ʹ | 127 |
| Pik3ca | F: 5ʹ- AGTGGCTCAAGGACAAGAACAAAGG-3ʹ  R: 5ʹ- GCCCAGTATAAAGGTAGCGACACAG-3ʹ | 101 |
| *Akt1* | F: 5ʹ- TCACTCCTCCTGACCAAGATGACAG-3ʹ  R: 5ʹ- GCGGTTCCACTGGCTGAATAGG-3ʹ | 94 |
| *Foxo3* | F: 5ʹ- TCAAGGACAAGGGCGACAACAAC-3ʹ  R: 5ʹ- GCTCTTCCCAGTGCCTTCGTTC-3ʹ | 113 |
| *P27* | F: 5ʹ- GAAGGCAGGTACGAGTGGCAAG-3ʹ  R: 5ʹ- GGTTTGGCAATTCCCGTTTACATCC-3ʹ | 135 |
| *P53* | F: 5ʹ- GCGGAGGAGATGGAACCATTGC-3ʹ  R: 5ʹ- GCTCCTGCCAGTTGCTGTGATC-3ʹ | 118 |
| Tsc1 | F: 5ʹ- AGCAGTAAAGGTGGCAGCAACAG-3ʹ  R: 5ʹ- ACAGTCAGTGGGACAGTAGTGGAG-3ʹ | 122 |
| mTOR | F: 5ʹ- AGCCTGCCTTATCCTCACCACTC-3ʹ  R: 5ʹ- TGGATTCGGTCATCACGGTTCATTC-3ʹ | 144 |
| Ulk1 | F: 5ʹ- GAGCAAGAGCACACCGACATCC-3ʹ  R: 5ʹ- TTTCAGGGCAGCAATCTCCATCAC-3ʹ | 84 |
| Ampk | F: 5ʹ- ACCATCTGTCTCGCCCTCATCC-3ʹ  R: 5ʹ- AATGCCACTTCGCTCTTCTTACACC-3ʹ | 132 |
| Pik3c3 | F: 5ʹ- GCTGAATAAGGAGATGGTGGAAGGC-3ʹ  R: 5ʹ- AGTTGGAGTACCTGCGGAGATGG-3ʹ | 110 |
| Lamp1 | F: 5ʹ- GAGACAAGTTTGGGGCAGTGGAAG-3ʹ  R: 5ʹ- GCGATCAGGACAATCAGAACCAGAC-3ʹ | 109 |
| *Mda5* | F: 5ʹ- GTGGCTTCAAGTGGCTCAGGAG-3ʹ  R: 5ʹ- AATACTCTTCTGGCGGCATCTCTTG-3ʹ | 112 |
| *Tlr21* | F: 5ʹ- TCTCACAGGCGGAGGTCTTCAC-3ʹ  R: 5ʹ- GCGAGGTTGGATGTCAGAGATGTC-3ʹ | 116 |
| *Nod1* | F: 5ʹ- CTGGTGCTGCTGGTAAGCCTTC-3ʹ  R: 5ʹ- CTCAGTTCCTGTCCGTGCTGTAAG-3ʹ | 81 |
| *Nlrp3* | F: 5ʹ- GCTCCTTGCGTGCTCTAAGACC-3ʹ  R: 5ʹ- TTGTGCTTCCAGATGCCGTCAG-3ʹ | 150 |
| *Nf-κb(p50)* | F: 5ʹ- GGTGGTATGTGGGAAGGCTTTGG-3ʹ  R: 5ʹ- CAGATGCTGGCTTTGTGATGTTGAC-3ʹ | 115 |
| *Il6* | F: 5ʹ- GAAATCCCTCCTCGCCAATCTGAAG-3ʹ  R: 5ʹ- GCCCTCACGGTCTTCTCCATAAAC-3ʹ | 108 |
| *Tnfα* | F: 5ʹ- CTCAGGACAGCCTATGCCAACAAG-3ʹ  R: 5ʹ- GGCGGTCATAGAACAGCACTACG-3ʹ | 86 |
| *cGAS* | F: 5ʹ- AAACACCTGGCGACTCTCTTTCTC-3ʹ  R: 5ʹ- TTCCTGCAACACTTCACTCCATCG-3ʹ | 105 |
| *Tbk1* | F: 5ʹ- TGGAATACAGAAGGCAGCAAACGG-3ʹ  R: 5ʹ- GCAAGGACAGGTGTGAGCAGTAC-3ʹ | 105 |
| *Sting* | F: 5ʹ- AGCTACTGGTCCTTGCCCTTGG-3ʹ  R: 5ʹ- CCGTGAGCGACATTCTTCTTGGAG-3ʹ | 91 |
| *Ifnα* | F: 5ʹ- TCCTTCAGAATACGGCTCCACCTC-3ʹ  R: 5ʹ- TGGCTGCTTGCTTCTTGTCCTTG-3ʹ | 90 |
| *Ifnβ* | F: 5ʹ- TCCTTCAGAATACGGCTCCACCTC-3ʹ  R: 5ʹ- TGGCTGCTTGCTTCTTGTCCTTG-3ʹ | 108 |
