# Supplementary material for: TMT-based quantitative proteomics analysis reveals the role of Notch signaling in FAdV-4-infected LMH cell
Source: Front Microbiol. 2022 Sep 15;13:988259. doi: 10.3389/fmicb.2022.988259 (PMC9520525; doi:10.3389/fmicb.2022.988259)
Supplement: Supplementary file 4 [file Data_Sheet_1.doc]

**Supplementary materials.**

**Table S1.** The differentially expressed proteins at 24 hpi comparing the experimental group and control group.

**Table S2.** Top 20 GO categories in the biological process, molecular function, and cellular component enriched at 24 hpi.
